# Extremum-Seeking Control for a Robotic Leg Prosthesis with Sensory Feedback

**DOI:** 10.3390/s25164975

**Published:** 2025-08-12

**Authors:** Ming Pi

**Affiliations:** School of Information and Control Engineering, Southwest University of Science and Technology, Mianyang 621000, China; pm198709@mail.ustc.edu.cn; Tel.: +86-15280986919; Fax: +86-0816-6089312

**Keywords:** sensory feedback, extremum-seeking control, robotic leg prosthesis

## Abstract

By sensing changes in the contact force between the leg and level ground, humans can perceive their walking speed and adjust leg stiffness to accommodate walking terrains. To realize this natural regulation mechanism on the lower limb amputee, noninvasive functional electrical stimulation (nFES) was used to assist the subject in sensing the change in contact force between the leg and level ground, allowing for the adjustment of control parameters in the prosthetic leg. The cost function was designed to combine the tracking errors of the joints and changes in the stimulating current. For different walking terrains, an extremum-seeking control (ESC) method was employed to search for suitable control parameters in real time by monitoring the changes in the cost function. The stability of the proposed controller with extremum-seeking dynamics was demonstrated. The experimental results demonstrated that the extremum-seeking method effectively adjusted the control parameters of the prosthetic leg in response to changes in the cost function.

## 1. Introduction

To realize the sense of the leg’s heel-strike moment and leave-off moment, noninvasive Functional Electrical Stimulation (nFES) was used to assist the subject in sensing changes in the contact force [1]. The stimulation current was proportional to the contact force. When the contact force changes significantly, the stimulation current also changed significantly, affecting the comfort of wearing the prosthesis [2]. To smooth the changes in the stimulation current and achieve excellent tracking performance, suitable control parameters for leg prostheses should be sought.

Functional Electrical Stimulation (FES) was widely used to reconstruct the sensations of prostheses. In [3], FES was adopted to help the subject sense the joint rotation of the prosthesis. The authors established a sensory feedback loop using FES to feed back the movement of the leg to humans in real time. However, the parameters of the FES were fixed and could not be adaptively regulated for walking terrain. In [4], FES was used to stimulate the limb muscles to induce limb movement through the brain–computer interface of the subject. The authors quantitatively compared desynchronization patterns during active, passive, and FES-induced movement of the lower limb. However, the sensory feedback induced by FES could not directly influence lower-limb movement. In [5], FES was used to stimulate the subjects’ lower limbs before their imagination to make them experience the contraction of muscles and improve their attention on the lower limbs by which it was supposed that the subjects’ motor imagery (MI) abilities could be enhanced. The authors combined FES and VR to reduce the difficulties in performing MI tasks and improve classification accuracy. However, the use of FES was simple and not for the control loop of the joint motor. Integrating the change in the FES to tune the control parameter of the leg prosthesis remains a challenge. Normally, by sensing the time difference between the leg’s heel-strike and leave-off moments, humans could perceive their walking speed and regulate leg stiffness to achieve a smooth change in the contact force [6]. In [7], the sensation of the contact force between the leg prosthesis and ground was restored. The authors introduced and tested a new type of neural prosthetic device and found that this prosthetic equipped with FES could help users improve walking speed and confidence by providing sensory information while reducing mental and physical fatigue as well as phantom limb pain. However, the impedance parameters of the leg prosthesis were predefined. The prosthesis could not tune the impedance parameters according to changes in the stimulation current.

In fact, it was important for the leg prosthesis to adaptively regulate the control parameters. In [8], a control method was modeled based on unified data-driven impedance control. The authors collected a kinematic dataset of eight normal humans to regulate impedance parameters for different walking conditions. However, collecting experimental data from different human models with varying support requirements was tedious and time-consuming. In [9], the control parameters were adjusted for different tasks. To reduce the response time of the device, the authors designed two velocity controllers and compared them with the native one. However, it was difficult to achieve a faster response owing to its nonlinear nature. In [10], motor control parameters were produced from able-bodied data and could be regulated. The authors presented a novel human–robot collaborative control scheme that achieves control weight self-adjustment for robotic prostheses to minimize the interaction torque. However, collecting kinematic datasets from different human models was tedious and time-consuming. In [11], an adaptive stair ascent controller for an above-knee leg prosthesis was discussed. With the adaptation to the changed heights of different stairs, the amputee can climb stairs with preferred cadence and gait patterns. The authors demonstrated the feasibility of adaptive dynamic programming (ADP) for automatic tuning of the 12 knee joint impedance parameters during a complete gait cycle to achieve balanced walking. However, the control parameters were adjusted based on the experience of the amputee.

To reduce the control parameters, a continuous-phase variable was presented for the motor control of the leg joints [12]. The motion of the knee and ankle joints could be parameterized using the same phase variable. There was a kinematic relationship between the angle of the knee joint and that of the ankle joint, which varied with the phase variable. In [13], the authors developed a method for calculating joint angles from digital snapshots or videos using computer vision and machine learning techniques to achieve a more accurate evaluation of ergonomic risk. Starting with an ergonomic analysis, this study explored the use of a semi-supervised training method to detect the skeletons of workers and estimate the positions and angles of their joints. However, the kinematic relationship between the joints has not yet been demonstrated. The motion parameters of each joint were independent. In [14], the authors presented a continuous-phase variable for stance phase control and an impedance control method for swing phase control. However, a method for designing the continuous-phase variable was required to collect a large amount of data on human movement. In [15], the authors introduced virtual constraints for powered prosthesis control, including steady-state and transitional gait. Kinematic interpolation was introduced to achieve a smooth transition between two steady states. However, the control parameters were trained using human motion data and could not be adaptively regulated. In [16], the kinematics of leg joints were parameterized under different walking speeds and inclines. They compared continuous-phase control with finite-state machine control. However, to provide motion accuracy, collecting motion data for different human bodies remains quite complex. In [17], the authors proposed a continuous-phase variable for the entire gait cycle. The unified control method reduced the parameters required for the motor control of the joints. However, the parameters for continuous-phase control were pre-trained and could not be automatically adjusted according to the various walking tasks.

To adaptively control the motion of the leg prosthesis, a cost function was typically designed to tune the control parameters [18]. In [19], the authors designed a cost function based on the metabolic cost of the subject to tune the joint control parameters of the exoskeleton. However, a longer optimization process was required to use the subject’s metabolism as the cost function. In [20], a data-driven approach based on a continuous-phase variable was used during the stance phase, whereas kinematic control was applied during the swing phase. The authors designed a cost function to optimize the impedance parameters under different walking conditions. However, the proposed optimization method required solving second-order differential equations and a relatively long optimization process. In [21], different impedance controllers were used and compared to reduce the subject’s metabolic cost. The authors designed a cost function to balance the system weight and assistance efficiency. However, the search process for optimizing the cost function usually required several hours [21]. In [22], the authors presented a human-in-the-loop (HIL) methodology that optimized the assistive torques provided by a powered hip exoskeleton. However, experiments have shown that the cost function based on metabolism was not suitable for real-time adaptation.

Therefore, an effective seeking method was required to achieve the real-time adaptation of the combined cost function [23]. In [24], the authors proposed an extremum-seeking method to determine the minimum cost function. This method was used to tune the stiffness of the leg prosthesis to minimize the cost function in real time. However, the initial parameters were manually set based on experience. In [25], an extremum-seeking method was developed to determine the optimal equilibrium of the cost function. The authors proved that the closed-loop extremum-seeking method could practically stabilize the unknown equilibrium optimum. In [26], the authors proposed an extremum-seeking controller for simultaneously tuning the feedback control gains of a knee–ankle-powered prosthetic leg using continuous-phase controllers. The developed cost function is then minimized using the extremum-seeking method in real time to simultaneously tune the proportional gains of the knee and ankle joints. However, the designed cost function could not reflect changes in the contact force and walking gait. In [27], the extremum-seeking method was employed to adapt the gains of a proportional–integral–derivative (PID) control law for functional neuromuscular electrical stimulation. The authors eliminated the initial tuning tests with patients because the controller parameters were automatically computed in real time. However, the control strategy was relatively simple and could not cope with the complex tracking performance.

The main motivation of this study is to optimize the control parameters to minimize the overall cost function of the system under different walking terrains. The cost function combines the joint tracking errors and changes in the stimulation current. The nFES is used to assist the subject in sensing changes in the contact force of the leg prosthesis. The stimulation current is proportional to the contact force. To incorporate both the tracking performance of the joints and the user comfort resulting from the stimulation current, a combined cost function is designed. An extremum-seeking method is proposed to adaptively regulate the control parameters to minimize the cost function [28]. The main innovation lies in the combination of the extremum-seeking method with adaptive control. The experimental results demonstrates that suitable control parameters for the leg prosthesis are found using the extremum-seeking method, achieving a balance between the tracking performance and changes in the stimulation current for different walking tasks.

The major contributions of this paper are outlined as follows: (1) nFES is used to assist the subject in sensing changes in contact force, and an observer is built to estimate the contact force; (2) the stimulation current influences the control parameters and the designed cost function considering the tracking errors of the joints and stimulating current. By minimizing the cost function, suitable control parameters are determined using the extremum-seeking method.

## 2. Materials and Methods

### 2.1. Dynamics of the Leg Prosthesis

**Figure 1:**
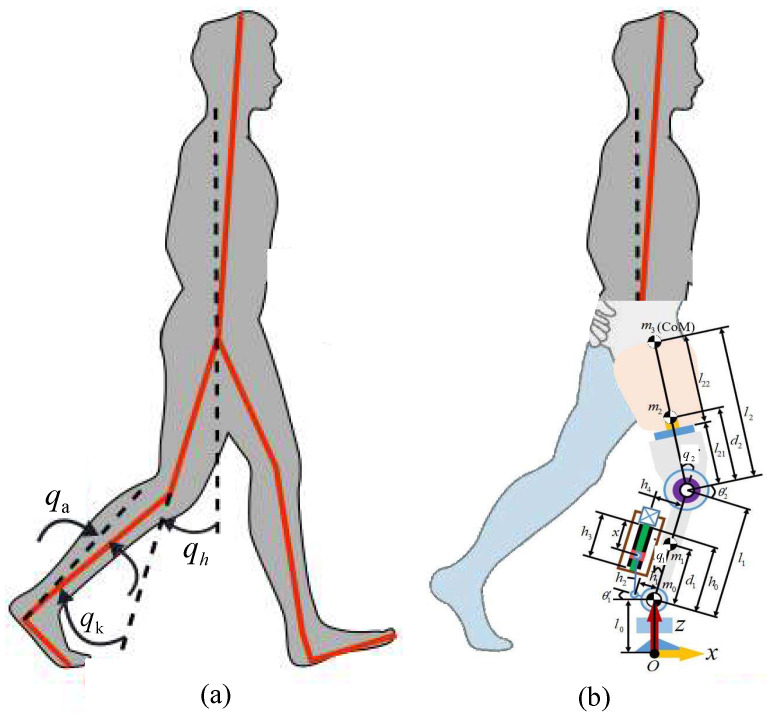
Definition of joint angles. (**a**) Able body. (**b**) Disabled body with prosthetic leg.

The 2-DOF powered robotic leg prosthesis features both an active ankle joint and an active knee joint. As shown in Figure 1, $q_{1}$ denotes the angle of the ankle joint, $q_{2}$ denotes the angle of the knee joint, $l_{1}$ denotes the distance from the ankle joint to the knee joint, and $l_{2}$ denotes the distance from the knee joint to the mass center of the human body. We assume that τ denotes the control torques for the leg joints, and $\tau_{hum}$ denotes the torque deduced from the contact force between the stump and the robotic leg: $\tau ={\left[\tau_{1},\tau_{2}\right]}^{T}$, and $\tau_{hum}={\left[\tau_{hum,1},\tau_{hum,2}\right]}^{T}$. $\tau_{1}$ represents the motor torque on the ankle joint of prosthesis. $\tau_{hum,1}$ represents the torque on the ankle joint of prosthesis from the contact force. $\tau_{2}$ represents the motor torque on the knee joint of the prosthesis. $\tau_{hum,2}$ represents the torque on the knee joint of prosthesis from the contact force.

Then, the dynamics of the leg prosthesis in the joint space can be written as

(1) $$\begin{array}{c} M_{q}\ddot{q}+C_{q}\dot{q}+G_{q}=\tau +\tau_{hum} \end{array}$$

where $q={\left[q_{1},q_{2}\right]}^{T}\in R^{2}$ denotes the real angle of joints, $M_{q}\in R^{2\times 2}$ denotes the inertia matrix, $M_{q}=\left[\begin{array}{cc} M_{11} & M_{12}\\ M_{21} & M_{22} \end{array}\right]$, $M_{11}=m_{1}d_{1}^{2}+I_{1}+I_{2}+m_{2}\left(l_{1}^{2}+d_{2}^{2}+2l_{1}l_{2}\cos q_{2}\right)$, $M_{12}=m_{2}d_{2}^{2}+I_{2}+m_{2}l_{2}d_{2}\cos q_{2}$, $M_{21}=m_{2}d_{2}^{2}+I_{2}+m_{2}l_{2}d_{2}\cos q_{2}$, $M_{22}=m_{2}d_{2}^{2}+I_{2}$; $C_{q}\in R^{2\times 2}$ is the centripetal–Coriolis matrix, $C_{q}=\left[\begin{array}{cc} C_{11} & C_{12}\\ C_{21} & C_{22} \end{array}\right]$, $C_{11}=-m_{2}l_{1}d_{2}\sin q_{2}{\dot{q}}_{2}$, $C_{12}=-m_{2}l_{1}d_{2}\sin q_{2}\left({\dot{q}}_{1}+{\dot{q}}_{2}\right)$, $C_{21}=m_{2}l_{1}d_{2}\sin q_{2}{\dot{q}}_{1}$, $C_{22}=0$; $G_{q}\in R^{2}$ is the gravitational torque, $G_{q}=\left[\begin{array}{c} m_{1}d_{1}g\cos q_{1}+m_{2}g\left(d_{2}\cos\left(q_{1}+q_{2}\right)+l_{1}\cos q_{1}\right)\\ m_{2}d_{2}g\cos\left(q_{1}+q_{2}\right) \end{array}\right]$. In these equations, $m_{i}$ is the mass of the *i*-th limb, $l_{i}$ is the length of the *i*-th limb, $d_{i}$ is the distance from the joint to the center of the *i*-th limb, $I_{i}$ is the moment of inertia of the *i*-th limb, *g* is gravity, and *i* = 1, 2.

The parameters of leg prosthesis can be designed as $m_{1}=1.5$ kg, $m_{2}=3$ kg, $d_{1}=$ 0.15 m, $d_{2}=$ 0.15 m, $I_{1}=1.3$ $\mathrm{kg}\cdot m^{2}$, $I_{2}=1.9$ $\mathrm{kg}\cdot m^{2}$, $l_{1}=0.222$ m, and $l_{2}=0.173$ m.

### 2.2. Restoration of Sensory Feedback

**Figure 2:**
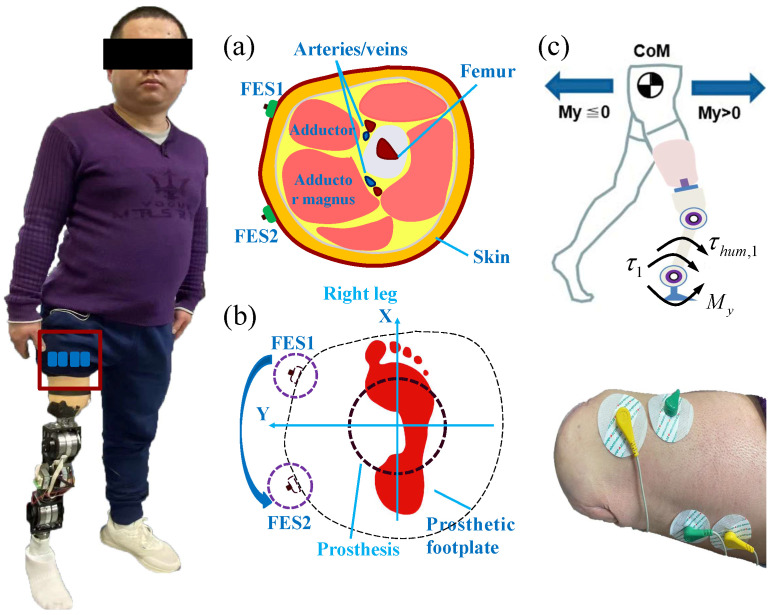
Sense the change of contact force of leg prosthesis. (**a**) Transversal surface of the stump of amputee. (**b**) Position of electrodes to induce the sense of torque $M_{y}$. (**c**) Relationship between $M_{y}$ and the motion of body.

To restore the sensation of the leg contact force while walking on the ground, two pairs of noninvasive electrodes were added to the amputee’s stump. As shown in Figure 2a, two electrodes FES1 and FES2 were pasted on the surface of the stump to stimulate the different muscles of the stump. As shown in Figure 2b, the sensation of the torque $M_{y}$ can be induced by the difference in the stimulating current of the two electrodes. $M_{y}$ denotes the total torque applied to the ankle joint. As shown in Figure 2c, the motion intention of the subject can be identified by a change in $M_{y}$. To align the direction of change of $M_{y}$ with the direction of the restored sensation, the electrodes’ positions were tested by feedback from the subject. The electrode for FES1 was positioned at the front side of the stump, and the target muscle group was the adductor muscle. The electrode for FES2 was positioned at the back side of the stump, and the target muscle group is the adductor magnus muscle. The stimulating current of FES1 and FES2 has the proportional relationship with torque $M_{y}$ according to (2) and (3). The relationship between the stimulating current of FES1 and the stimulating current of FES2 and $M_{y}$ can be expressed as

(2) $$\begin{array}{c} FES1=k_{FES1}M_{y},\mathrm{when}\;M_{y}>0 \end{array}$$

(3) $$\begin{array}{c} FES2=-k_{FES2}M_{y},\mathrm{when}\;M_{y}\leq 0 \end{array}$$

where $k_{FES1}$ and $k_{FES2}$ denote the positive constants. When $M_{y}>0$, it means that the CoM of the whole body is at the front of the leg prosthesis; the stimulating current of FES1 changes more to induce the sensation for the changed position of CoM; when $M_{y}\leq 0$, it means that the CoM of the whole body is at the back of the leg prosthesis; the stimulating current of FES2 changes to induce the sensation for the changed position of CoM. The sensations of the leg’s heel-strike moment and leave-off moment were modeled as $\Gamma_{sense}={\left[T_{heel-strike},\;T_{leave-off}\right]}^{T}=H{\left[FES1,\;FES2\right]}^{T}$, where $\Gamma_{sense}$ denotes the restored sensation, $T_{heel-strike}$ denotes the sensation for the heel-strike moment, $T_{leave-off}$ denotes the sensation for the leave-off moment, and $H=\left[\begin{array}{cc} 5 & 0\\ 0 & 5 \end{array}\right]\in R^{2\times 2}$ denotes the constant matrix. As shown in Figure 2c, $M_{y}$ can be computed as

(4) $$\begin{array}{c} M_{y}=-\tau_{1}-\tau_{hum,1} \end{array}$$

Assuming the estimation of $\tau_{hum,1}$ as ${\hat{\tau }}_{hum,1}$, we have $M_{y}=-\tau_{1}-{\hat{\tau }}_{hum,1}$ Then, we have

(5) $$\begin{array}{c} F_{contact}=\Upsilon^{-1}\left(\tau +\tau_{hum}\right) \end{array}$$

where τ and $\tau_{hum}$ will be analyzed later; $F_{contact}={\left[F_{x},F_{z}\right]}^{T}$ represents the contact force between the ground and the robotic leg; Υ represents the Jacobian matrix, $\Upsilon =\left[\begin{array}{cc} -l_{1}\sin q_{1}-l_{2}\sin\left(q_{1}+q_{2}\right) & l_{1}\cos q_{1}+l_{2}\cos\left(q_{1}+q_{2}\right)\\ -l_{2}\sin\left(q_{1}+q_{2}\right) & l_{2}\cos\left(q_{1}+q_{2}\right) \end{array}\right]$. When the change of contact force is stiff, causing the drastic change of the stimulating current, and affecting the comfort of walking, $\tau_{hum}$ become bigger simultaneously [29].

### 2.3. Continuous-Phase Variable

In Figure 1a, the subject’s thigh angle $q_{h}$ is synchronized with the motion of the knee and ankle joints of the leg $q_{k}$ and $q_{a}$. Hence, the desired ankle and knee trajectories $q_{d}={\left[q_{1d},q_{2d}\right]}^{T}$ of the prosthesis leg joints can also be generated from $q_{h}$. $q_{h}$ denotes the thigh angle of the subject and can be measured by the sensor.

The phase variable ν∈[0,1) was introduced to parameterize the subject’s thigh angle $q_{h}$. The trajectory of $q_{h}$ during one gait consisted of two parts: descending and ascending. After the heel-strike moment, the trajectory of $q_{h}$ decreased during the stance phase. When the stance phase reaches its minimum value, $q_{h}$ increased. The descending part corresponds roughly to the stance phase, whereas the ascending part corresponds roughly to the swing phase. The phase variable ν can be calculated from $q_{h}$ as

(6) $$\begin{array}{c} \nu =\left\{\begin{array}{cc} w_{stance}\left(q_{h}^{0}-q_{h}\right), & Stance\;phase\\ 1+w_{swing}\left(q_{h}^{0}-q_{h}\right), & Swing\;phase \end{array}\right. \end{array}$$

where $w_{stance}=\frac{\nu_{m}}{q_{h}^{0}-q_{h}^{min}}$, $w_{swing}=\frac{\nu_{m}-1}{q_{h}^{0}-q_{h}^{min}}$, and $q_{h}^{0}$, $q_{h}^{min}$, and $\nu_{m}$ are 0 degree, −20 degree, and 0.6, respectively. According to (6), ν is limited from 0 to 1. Then, we obtain $q_{d}$ as [12]

(7) $$\begin{array}{cc} q_{d}= & \frac{1}{2}\rho_{0}+\frac{1}{2}\rho_{N}cos\left(\pi N\nu \right)\\ \; & +\sum_{k=1}^{N-1}\left[\rho_{k}cos\left(2\pi k\nu \right)-\Psi_{k}sin\left(2\pi k\nu \right)\right] \end{array}$$

where $\rho_{0}$, $\rho_{N}$, $\rho_{k}$, and $\Psi_{k}$ are 12.5, 8.9, 10.6, and 7.5, respectively, which were calculated from human walking data. *N* denotes the total order of the Fourier series, and N=4. *k* denotes the *k*-th order of Fourier series.

### 2.4. Control Development

#### 2.4.1. Controller Design

The controller for the joints of leg prosthesis can be designed as

(8) $$\begin{array}{c} \tau =M_{q}\left({\ddot{q}}_{r}-\kappa_{2}s\right)+C_{q}{\dot{q}}_{r}+G_{q}-{\hat{\tau }}_{hum} \end{array}$$

where $s=\dot{e}+\kappa_{1}e$, $e=q-q_{d}$, ${\dot{q}}_{r}={\dot{q}}_{d}-\kappa_{1}e$, *e* denotes the tracking error of joints, $\kappa_{1}$ and $\kappa_{2}$ denote the positive constants, and ${\hat{\tau }}_{hum}$ denotes the estimation of $\tau_{hum}$.

Substituting (8) into (1), we have

(9) $$\begin{array}{c} M_{q}\left(\ddot{q}-{\ddot{q}}_{r}+\kappa_{2}s\right)+C_{q}\left(\dot{q}-{\dot{q}}_{r}\right)+{\hat{\tau }}_{hum}-\tau_{hum}=0 \end{array}$$

For $s=\dot{e}+\kappa_{1}e$, $e=q-q_{d}$, and ${\dot{q}}_{r}={\dot{q}}_{d}-\kappa_{1}e$, we have

(10) $$\begin{array}{c} M_{q}\left(\dot{s}+\kappa_{2}s\right)+C_{q}s+{\hat{\tau }}_{hum}-\tau_{hum}=0 \end{array}$$

Assuming ${\tilde{\tau }}_{hum}={\hat{\tau }}_{hum}-\tau_{hum}$, we have

(11) $$\begin{array}{c} M_{q}\dot{s}=-\kappa_{2}M_{q}s-C_{q}s-{\tilde{\tau }}_{hum} \end{array}$$

Moreover, (11) can be deduced as

(12) $$\begin{array}{c} \dot{s}=-\kappa_{2}s-M_{q}^{-1}C_{q}s-M_{q}^{-1}{\hat{\tau }}_{hum}+M_{q}^{-1}\tau_{hum} \end{array}$$

The initial value of $\tau_{hum}$ can be computed using (1). To construct the observer for ${\hat{\tau }}_{hum}$, the adaptation law is defined as

(13) $$\begin{array}{c} {\dot{\hat{\tau }}}_{hum}=-\Gamma_{\tau }\hat{K}\left(T_{1}+\alpha T_{2}\right) \end{array}$$

where $\Gamma_{\tau }$ and α are positive constants. ${\hat{\tau }}_{hum}={\hat{\tau }}_{hum,pre}+{\dot{\hat{\tau }}}_{hum}$, and ${\hat{\tau }}_{hum,pre}$ denotes the pre-value of ${\hat{\tau }}_{hum}$. Inspired by [30], the two variables $T_{1}$ and $T_{2}$ were designed as follows:

(14) $$\begin{array}{cc} \; & T_{1}=-F_{1}{\tilde{\tau }}_{hum}+\upsilon_{2} \end{array}$$

(15) $$\begin{array}{cc} \; & T_{2}=-{M_{q}^{-1}}^{T}M_{q}^{-1}{\tilde{\tau }}_{hum} \end{array}$$

where $\upsilon_{2}=\int_{0}^{t}e^{-c(t-r)}{M_{q}^{-1}}^{T}M_{q}^{-1}\left(\tau_{hum}\left(t\right)-\tau_{hum}\left(r\right)\right)dr$ [31]. To simplify the analysis, we have

(16) $$\begin{array}{cc} \; & T_{1}=-F_{1}{\hat{\tau }}_{hum}-F_{2} \end{array}$$

(17) $$\begin{array}{cc} \; & T_{2}=-{M_{q}^{-1}}^{T}M_{q}^{-1}{\hat{\tau }}_{hum}+{M_{q}^{-1}}^{T}M_{q}^{-1}\tau_{hum} \end{array}$$

The adaptation laws for $F_{1}$ and $F_{2}$ are designed as [30]

(18) $$\begin{array}{c} {\dot{F}}_{1}=-cF_{1}+{M_{q}^{-1}}^{T}M_{q}^{-1} \end{array}$$

(19) $$\begin{array}{c} {\dot{F}}_{2}=-cF_{2}+{M_{q}^{-1}}^{T}M_{q}^{-1}\tau_{hum} \end{array}$$

where *c* denotes the positive gain. The time integration of (18) and (19) results in

(20) $$\begin{array}{cc} \; & F_{1}=\int_{0}^{t}e^{-c(t-r)}{M_{q}^{-1}}^{T}M_{q}^{-1}dr \end{array}$$

(21) $$\begin{array}{cc} \; & F_{2}=\int_{0}^{t}e^{-c(t-r)}{M_{q}^{-1}}^{T}M_{q}^{-1}\tau_{hum}dr \end{array}$$

#### 2.4.2. Design of Cost Function

The aim of the control method is to adaptively tune the control parameter $\hat{K}$ to achieve a balance between the tracking errors and change in the stimulating current [32]. Assuming $K_{dcmf}$ denotes the degree of the change of the stimulating current, and ${\hat{K}}_{dcmf}$ denotes the estimation of $K_{dcmf}$, we have

(22) $$\begin{array}{c} {\hat{K}}_{dcmf}=A\frac{\left|FES1|+|FES2\right|}{T_{gait}} \end{array}$$

where |FES1| and |FES2| can be computed from $M_{y}$ according to (2) and (3), respectively, $T_{gait}$ denotes the walking gait cycle. *A* is a positive constant. Hence, the cost function $J_{dcmf}$ for the change in the stimulating current can be designed as

(23) $$\begin{array}{c} J_{dcmf}=\frac{1}{{\left({\hat{K}}_{dcmf}-{\bar{\hat{K}}}_{dcmf}\right)}^{2}} \end{array}$$

where ${\bar{\hat{K}}}_{dcmf}$ denotes the upper bound of ${\hat{K}}_{dcmf}$, ${\bar{\hat{K}}}_{dcmf}$ = 1.35. Then, we obtain the combined cost function *J* as

(24) $$\begin{array}{cc} J & =\omega_{err}{∥e∥}^{2}+\omega_{dcmf}J_{dcmf}\\ \; & =\omega_{err}{∥e∥}^{2}+\omega_{dcmf}\frac{1}{{\left({\hat{K}}_{dcmf}-{\bar{\hat{K}}}_{dcmf}\right)}^{2}} \end{array}$$

where $\omega_{err}$ denotes the weighting factor for the tracking performance of joints, and $\omega_{dcmf}$ denotes the weighting factor for the change in the stimulating current and is designed as a constant value.

#### 2.4.3. Extremum-Seeking Method

**Figure 3:**
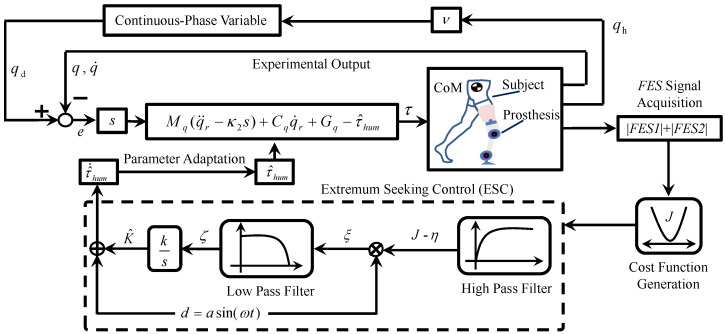
Diagram of the control flow. The input trajectory $q_{d}$ is generated from continuous-phase variable, and the output trajectory is *q*. With the extremum-seeking control process, ${\hat{\tau }}_{hum}$ was estimated to minimize the cost function *J*.

To minimize the combined cost function *J*, an extremum-seeking method is presented. As shown in Figure 3, the seeking signal is designed as d(t)=asin(ωt) to estimate $\hat{K}$. According to (13), $\hat{K}$ is the key parameter for the estimation ${\hat{\tau }}_{hum}$. According to (8), $\hat{K}$ influences the control performance of the joint’s motor control. Then, the cost function *J* is generated from (24). Using a high-pass filter (HPF), the DC component η was removed from *J*. Then, J−η was modulated by the seeking signal d(t). Using the low-pass filter (LPF) and integrator, $\hat{K}$ can be demodulated from ζ(t) by the same seeking signal d(t).

The extremum-seeking method searches for suitable control parameters $\hat{K}$ with a change in the cost function *J* [32]. The adaptive law of the extremum-seeking method can be expressed as

(25) $$\begin{array}{cc} \; & \dot{\hat{K}}=k\xi \end{array}$$

(26) $$\begin{array}{cc} \; & \dot{\xi }=-\omega_{l}\xi +\omega_{l}\left(J-\eta \right)a\sin\left(\omega t\right) \end{array}$$

(27) $$\begin{array}{cc} \; & \dot{\eta }=\omega_{h}\left(J-\eta \right) \end{array}$$

where $\omega_{l}$ and $\omega_{h}$ represent the cutoff frequencies for the low and high pass, respectively; $\hat{K}={\hat{K}}_{pre}+\dot{\hat{K}}$, $\xi =\xi_{pre}+\dot{\xi }$, and $\eta =\eta_{pre}+\dot{\eta }$. ${\hat{K}}_{pre}$ denotes the pre-value of $\hat{K}$; $\xi_{pre}$ denotes the pre-value of ξ; $\eta_{pre}$ denotes the pre-value of η.

As shown in Figure 3, the block diagram of the control method is depicted as three main components: (1) $q_{d}$ was generated from the phase variable ν; (2) τ was calculated from the controller to conduct the motor control of the leg prosthesis; and (3) an extremum-seeking method was proposed to search for the control parameter $\hat{K}$ to minimize the designed cost function *J*.

#### 2.4.4. Stability Analysis

To analyze the stability of the proposed controller, the Lyapunov function can be selected as

(28) $$\begin{array}{c} V\left(t\right)=\frac{1}{2}\left(s^{T}M_{q}s+\Gamma_{\tau }^{-1}{\tilde{\tau }}_{hum}^{T}\hat{K}{\tilde{\tau }}_{hum}\right) \end{array}$$

where $\Gamma_{\tau }$ denotes the positive constant scaling factor matrix. We then obtain the time derivative for (28) as

(29) $$\begin{array}{cc} \dot{V}\left(t\right)= & s^{T}M_{q}\dot{s}+\Gamma_{\tau }^{-1}{\tilde{\tau }}_{hum}^{T}\hat{K}{\dot{\tilde{\tau }}}_{hum}\\ \; & +\frac{1}{2}\left(s^{T}{\dot{M}}_{q}s+\Gamma_{\tau }^{-1}{\tilde{\tau }}_{hum}^{T}\dot{\hat{K}}{\tilde{\tau }}_{hum}\right) \end{array}$$

According to (12), we have

(30) $$\begin{array}{cc} \dot{V}\left(t\right)= & s^{T}M_{q}\left(-\kappa_{2}s-M_{q}^{-1}C_{q}s-M_{q}^{-1}{\hat{\tau }}_{hum}+M_{q}^{-1}\tau_{hum}\right)\\ \; & +\Gamma_{\tau }^{-1}{\tilde{\tau }}_{hum}^{T}\hat{K}\left({\dot{\hat{\tau }}}_{hum}-{\dot{\tau }}_{hum}\right)\\ \; & +\frac{1}{2}\left(s^{T}{\dot{M}}_{q}s+\Gamma_{\tau }^{-1}{\tilde{\tau }}_{hum}^{T}\dot{\hat{K}}{\tilde{\tau }}_{hum}\right)\\ = & -\kappa_{2}s^{T}M_{q}s-s^{T}C_{q}s-s^{T}{\tilde{\tau }}_{hum}\\ \; & +\Gamma_{\tau }^{-1}{\tilde{\tau }}_{hum}^{T}\hat{K}\left({\dot{\hat{\tau }}}_{hum}-{\dot{\tau }}_{hum}\right)\\ \; & +\frac{1}{2}s^{T}{\dot{M}}_{q}s+\frac{1}{2}\Gamma_{\tau }^{-1}{\tilde{\tau }}_{hum}^{T}\dot{\hat{K}}{\tilde{\tau }}_{hum} \end{array}$$

According to (13), we have

(31) $$\begin{array}{cc} \dot{V}\left(t\right)= & -\kappa_{2}s^{T}M_{q}s-s^{T}{\tilde{\tau }}_{hum}+\frac{1}{2}s^{T}\left({\dot{M}}_{q}-2C_{q}\right)s\\ \; & +\Gamma_{\tau }^{-1}{\tilde{\tau }}_{hum}^{T}\hat{K}(-\Gamma_{\tau }\hat{K}(-F_{1}{\tilde{\tau }}_{hum}+\upsilon_{2}\\ \; & -\alpha {M_{q}^{-1}}^{T}M_{q}^{-1}{\tilde{\tau }}_{hum})-{\dot{\tau }}_{hum})+\frac{1}{2}\Gamma_{\tau }^{-1}{\tilde{\tau }}_{hum}^{T}\dot{\hat{K}}{\tilde{\tau }}_{hum}\\ = & -\kappa_{2}s^{T}M_{q}s-s^{T}{\tilde{\tau }}_{hum}+\frac{1}{2}s^{T}\left({\dot{M}}_{q}-2C_{q}\right)s\\ \; & +{\tilde{\tau }}_{hum}^{T}\hat{K}\hat{K}F_{1}{\tilde{\tau }}_{hum}-{\tilde{\tau }}_{hum}^{T}\hat{K}\hat{K}\upsilon_{2}\\ \; & +{\tilde{\tau }}_{hum}^{T}\hat{K}\hat{K}\alpha {\tilde{\tau }}_{hum}-\Gamma_{\tau }^{-1}{\tilde{\tau }}_{hum}^{T}\hat{K}{\dot{\tau }}_{hum}\\ \; & +\frac{1}{2}\Gamma_{\tau }^{-1}{\tilde{\tau }}_{hum}^{T}\dot{\hat{K}}{\tilde{\tau }}_{hum} \end{array}$$

As the matrix ${\dot{M}}_{q}-2C_{q}$ is skew-symmetric, $s^{T}\left({\dot{M}}_{q}-2C_{q}\right)s=0$, we have

(32) $$\begin{array}{cc} \dot{V}\left(t\right)= & -\kappa_{2}∥M_{q}{∥∥s∥}^{2}-∥s∥∥{\tilde{\tau }}_{hum}∥\\ \; & +∥\hat{K}{∥}^{2}∥F_{1}∥∥{\tilde{\tau }}_{hum}{∥}^{2}-∥\hat{K}{∥}^{2}∥\upsilon_{2}∥∥{\tilde{\tau }}_{hum}^{T}∥\\ \; & +\alpha ∥\hat{K}{∥}^{2}∥{\tilde{\tau }}_{hum}{∥}^{2}-∥\Gamma_{\tau }^{-1}∥∥{\tilde{\tau }}_{hum}^{T}∥∥\hat{K}∥∥{\dot{\tau }}_{hum}∥\\ \; & +\frac{1}{2}∥\Gamma_{\tau }^{-1}∥∥\dot{\hat{K}}∥∥{\tilde{\tau }}_{hum}{∥}^{2} \end{array}$$

Then, $\dot{V}\left(t\right)$ can be formulated as

(33) $$\begin{array}{cc} \dot{V}\left(t\right)\leq & -\kappa_{2}\max(∥M_{q}{∥)∥s∥}^{2}-∥s∥∥{\tilde{\tau }}_{hum}∥\\ \; & +\min(∥\hat{K}{∥)}^{2}\min(∥F_{1}∥)∥{\tilde{\tau }}_{hum}{∥}^{2}\\ \; & -\max(∥\hat{K}{∥)}^{2}\max(∥\upsilon_{2}∥)∥{\tilde{\tau }}_{hum}^{T}∥\\ \; & +\alpha \min(∥\hat{K}{∥)}^{2}{∥{\tilde{\tau }}_{hum}∥}^{2}\\ \; & -\Gamma_{\tau }^{-1}∥{\tilde{\tau }}_{hum}^{T}∥\max(∥\hat{K}∥)\max(∥{\dot{\tau }}_{hum}∥)\\ \; & +\frac{1}{2}\Gamma_{\tau }^{-1}\max(∥\dot{\hat{K}}∥)∥{\tilde{\tau }}_{hum}{∥}^{2} \end{array}$$

Assuming that $M_{1}$ is the min-bound of $∥M_{q}∥$, $M_{1}$ = 0.9, $M_{2}$ is the max-bound of $∥M_{q}∥$, $M_{2}$ = 1.2, $R_{1}$ is the min-bound of $∥\hat{K}∥$, $R_{2}$ is the max-bound of $∥\hat{K}∥$, $\gamma_{2}$ is the maximum of $∥\upsilon_{2}∥$, $\rho_{\tau d}$ is the maximum of $∥{\dot{\tau }}_{hum}∥$, $\eta_{F}$ is the minimum of $∥F_{1}∥$, χ is the minimum of $\dot{\hat{K}}$, α, and *c*, and $\kappa_{2}$ are positive constants, respectively, we have

(34) $$\begin{array}{cc} \dot{V}\left(t\right)\leq & -\kappa_{2}M_{2}{∥s∥}^{2}-∥s∥∥{\tilde{\tau }}_{hum}∥\\ \; & +R_{1}^{2}\eta_{F}∥{\tilde{\tau }}_{hum}{∥}^{2}-R_{2}^{2}\gamma_{2}∥{\tilde{\tau }}_{hum}^{T}∥+\alpha R_{1}^{2}{∥{\tilde{\tau }}_{hum}∥}^{2}\\ \; & -\Gamma_{\tau }^{-1}∥{\tilde{\tau }}_{hum}∥R_{2}\rho_{\tau d}+\frac{1}{2}\Gamma_{\tau }^{-1}\chi {∥{\tilde{\tau }}_{hum}∥}^{2} \end{array}$$

Considering the limits of matrices, the limit of $\dot{V}\left(t\right)$ can be calculated as

(35) $$\begin{array}{cc} \dot{V}\left(t\right)\leq & -\hbar_{s}{∥s∥}^{2}-∥s∥∥{\tilde{\tau }}_{hum}∥\\ \; & +\hbar_{\tau 1}∥{\tilde{\tau }}_{hum}{∥}^{2}+\hbar_{\tau 2}∥{\tilde{\tau }}_{hum}∥ \end{array}$$

where

(36) $$\begin{array}{cc} \; & \hbar_{s}=\kappa_{2}M_{2} \end{array}$$

(37) $$\begin{array}{cc} \; & \hbar_{\tau 1}=R_{1}^{2}\eta_{F}+\alpha R_{1}^{2}+\frac{1}{2}\Gamma_{\tau }^{-1}\chi \end{array}$$

(38) $$\begin{array}{cc} \; & \hbar_{\tau 2}=-R_{2}^{2}\gamma_{2}-\Gamma_{\tau }^{-1}R_{2}\rho_{\tau d} \end{array}$$

Hence, we have the conclusion that $\dot{V}\left(t\right)<0$ when inside the compact region:

(39) $$\begin{array}{c} ∥s∥\leq \frac{-{ℜ}_{2}+\sqrt{{ℜ}_{2}^{2}+4{ℜ}_{1}{ℜ}_{3}}}{2{ℜ}_{1}} \end{array}$$

(40) $$\begin{array}{c} ∥{\tilde{\tau }}_{hum}∥\leq \frac{-{ℜ}_{5}+\sqrt{{ℜ}_{5}^{2}+4{ℜ}_{4}{ℜ}_{6}}}{2{ℜ}_{4}} \end{array}$$

where ${ℜ}_{1}=\hbar_{s}$, ${ℜ}_{2}=∥{\tilde{\tau }}_{hum}∥$, ${ℜ}_{3}=\hbar_{\tau 2}∥{\tilde{\tau }}_{hum}∥$, ${ℜ}_{4}=\hbar_{\tau 1}$, ${ℜ}_{5}=∥s∥+{ℜ}_{\tau 2}$, and ${ℜ}_{6}=\hbar_{s}$.

From the above analysis, the estimate of $\tau_{hum}$ also converges inside this compact region. Moreover, owing to the limits of the tracking dynamics *s* and adaptive law ${\dot{\hat{\tau }}}_{hum}$, the Lyapunov function candidate can be bounded by the limits of (39) and (40). Hence, the uniformly ultimately bounded (UUB) stability of the controller can be guaranteed.

## 3. Experiments and Results

### 3.1. Experiment Setup

Walking speed and slope are two important factors that affect human walking performance. Hence, four cases with different walking speeds and slopes were considered to verify the effectiveness of the proposed approach. To maintain objectivity and consistency between different cases, all other conditions were kept the same, except for walking speed and slope.

#### 3.1.1. Study Volunteers

Three subjects were recruited for this study. Both the subjects were tested by a psychologist beforehand and agreed to participate in the experiments. They understood all the experimental procedures and singed informed consent forms. Subject 1 was 33 years old and had a right leg stump for 4 years. Subject 2 was 34 years old and had a right leg stump for 5 years. Subject 3 was 22 years old and had a right leg stump for 2 years. All experimental procedures were approved by the Ethics Committee of the Yueyang Hospital of Integrated Traditional Chinese and Western Medicine, Shanghai University of Traditional Chinese Medicine. The experiments were registered at the China Clinical Trial Registration Center, numbered as ChiCTR2000031162. The protocol was authorized, numbered as 2019-014.

#### 3.1.2. Robotic Leg Prosthesis

Figure 1 shows a sketch of the leg prosthesis. The leg prosthesis consists of a mechanical structure, control system, and sensor system. Table 1 lists the mass weights of the leg prostheses.

### 3.2. Case1: Assessment of Walk from Low Speed to High Speed

#### 3.2.1. Experiment

In this experiment, the subjects were required to walk from low speed 0.5 ± 0.1 m/s to high speed 1.0 ± 0.1 m/s. In the first 10 s, the walking speed was maintained at 0.5 ± 0.1 m/s. Then, the subject walk from low speed 0.5 ± 0.1 m/s to middle speed 0.7 ± 0.1 m/s for 9 s. Finally, the walking speed was maintained at 1.0 ± 0.1 for 10 s.

#### 3.2.2. Results

**Figure 4:**
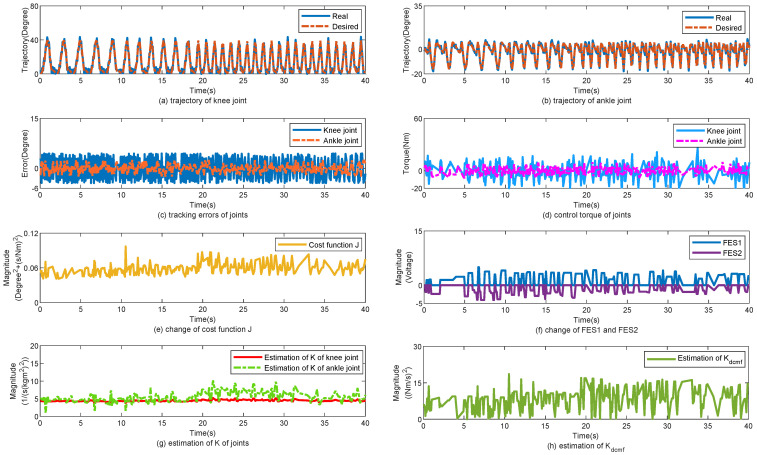
Walk from low speed to high speed. (**a**) Tracking performance on knee joint. (**b**) Tracking performance on ankle joint. (**c**) Tracking errors of joints. (**d**) Control torque of joints. (**e**) Change of cost function J. (**f**) Change of FES1 and FES2. (**g**) Estimation of K of joints. (**h**) Estimation of $K_{dcmf}$.

The control performance of the proposed method is displayed in Figure 4. Figure 4a,b show the tracking trajectories of the knee and ankle joints, respectively. In the first 10 s, the walking gait cycle was approximately 2 s, and the walking speed was approximately 0.5 m/s. The gait cycle was changed to 1.5 s for 9 s. Finally, the gait cycle was maintained at 1 s. Figure 4c shows the tracking errors of the knee and ankle joints, respectively. Despite the change in the walking speed, the tracking errors of the joints did not change significantly. Figure 4d shows the changes in the torques of the knee and ankle joints. Figure 4e shows the change in cost function *J*. According to (22) and (24), *J* is related to the gait cycle $T_{gait}$. When $T_{gait}$ was changed from 2 to 1 s, *J* increased simultaneously. Figure 4f shows the changes in the FES1 and FES2. Figure 4g,h show the changes in $\hat{K}$ and ${\hat{K}}_{dcmf}$, respectively. $\hat{K}$ and ${\hat{K}}_{dcmf}$ change simultaneously with $T_{gait}$. In Table 2, the tracking errors are statistically calculated for the three subjects. With the help of the proposed control method, the mean error (MEAN) of the tracking performance is 3.6°, 3.3°, and 3.4° and the minimized mean square errors (MSE) of the tracking performance are 2.3°, 2.8°, and 2.75°, respectively.

### 3.3. Case2: Assessment of Walk from High Speed to Low Speed

#### 3.3.1. Experiment

In this experiment, the subjects were required to walk from high speed 1.0 ± 0.1 m/s to low speed 0.5 ± 0.1 m/s. In the first 10 s, the walking speed was maintained at 1.0 ± 0.1 m/s. Then, the subject walk from high speed 1.0 ± 0.1 m/s to middle speed 0.7 ± 0.1 m/s for 9 s. At last, the walking speed was maintained at 0.5 ± 0.1 m/s for 10 s.

#### 3.3.2. Results

**Figure 5:**
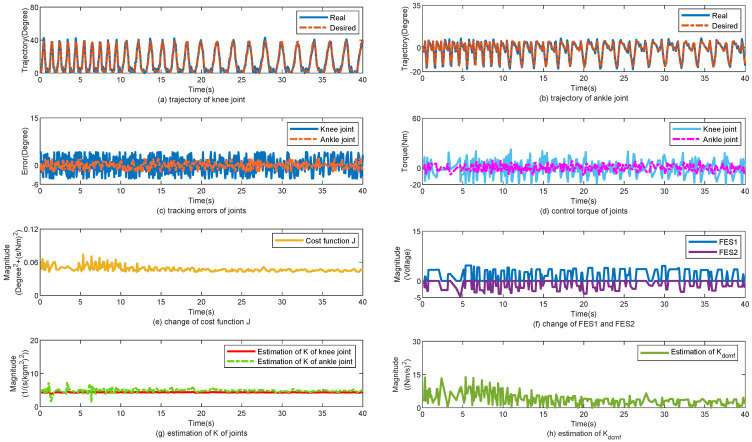
Walk from high speed to low speed. (**a**) Tracking performance on knee joint. (**b**) Tracking performance on ankle joint. (**c**) Tracking errors of joints. (**d**) Control torque of joints. (**e**) Change of cost function J. (**f**) Change of FES1 and FES2. (**g**) Estimation of K of joints. (**h**) Estimation of $K_{dcmf}$.

The control performance of the proposed method is displayed in Figure 5. Figure 5a,b show the tracking trajectories of the knee and ankle joints, respectively. During the first 10 s, the walking gait cycle was approximately 1 s, and the walking speed was approximately 1.0 m/s. The gait cycle was changed to 1.5 s for 9 s. Finally, the gait cycle was maintained for 2 s. Figure 5c shows the tracking errors of the knee and ankle joints. Despite the change in the walking speed, the tracking errors of the joints did not change significantly. Figure 5d shows the change in the torques of the knee and ankle joints. Figure 5e shows the change in cost function *J*. According to (22) and (24), *J* is related to the gait cycle $T_{gait}$. When $T_{gait}$ was changed from 1 to 2 s, *J* decreased simultaneously. Figure 5f shows the changes in FES1 and FES2. Figure 5g,h show the changes in $\hat{K}$ and ${\hat{K}}_{dcmf}$, respectively. $\hat{K}$ and ${\hat{K}}_{dcmf}$ changes simultaneously with $T_{gait}$. In Table 3, the tracking errors are statistically calculated for the three subjects. With the help of the proposed control method, the mean error (MEAN) of the tracking performance is 2.7°, 3.1°, and 3.2°, respectively; the minimized mean square errors (MSE) of the tracking performance are 1.8°, 2.4°, and 2.3°, respectively.

### 3.4. Case3: Assessment of Walk from Level Ground to Up Slope

#### 3.4.1. Experiment

In this experiment, the subjects were required to walk from level ground to different slopes. The slope angle was varied from 3° to 5° and 7°, respectively. In the first 20 s, the subject walked at ground level when the slope angle was 3°. Then, the slope angle gradually changed from 3° to 5° and then to 7°, each lasting for 20 s. The gait cycle was maintained for 2 s.

#### 3.4.2. Results

**Figure 6:**
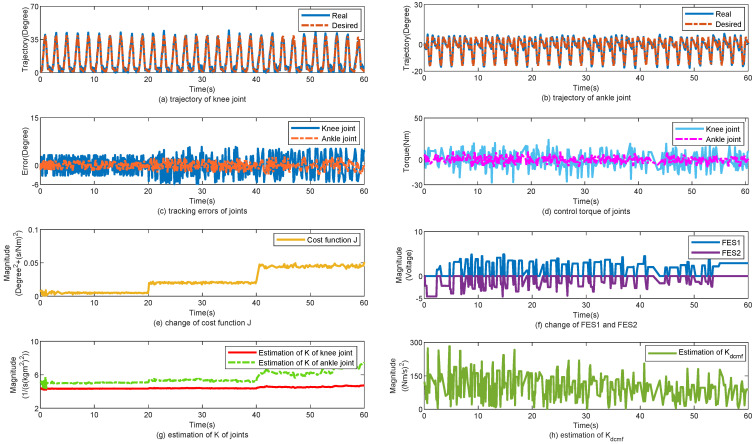
Walk from level ground to up slop. (**a**) Tracking performance on knee joint. (**b**) Tracking performance on ankle joint. (**c**) Tracking errors of joints. (**d**) Control torque of joints. (**e**) Change of cost function J. (**f**) Change of FES1 and FES2. (**g**) Estimation of K of joints. (**h**) Estimation of $K_{dcmf}$.

The control performance of the proposed method is displayed in Figure 6. Figure 6a,b show the tracking trajectories of the knee and ankle joints, respectively. In the first 20 s, the slope angle was approximately 3°, and the gait cycle was approximately 2 s. The slope angle was then increased to 5° for 20 s. Finally, the slope angle was maintained at 7°. Figure 6c shows the tracking errors of the knee and ankle joints. With an increase in the slope angle, the load for the joints increased, and the tracking errors of the joints changed simultaneously. Figure 6d shows the change in the torques of the knee and ankle joints. Figure 6e shows the change in cost function *J*. According to (24), *J* is related to the tracking errors of joint *e*. When *e* is changed, *J* changes simultaneously. Figure 6f shows the change in FES1 and FES2. Figure 6g,h show the changes in $\hat{K}$ and ${\hat{K}}_{dcmf}$, respectively. $\hat{K}$ increased simultaneously with *J* . ${\hat{K}}_{dcmf}$ decreased with *J*. In Table 4, the tracking errors are statistically calculated for the three subjects. With the help of the proposed control method, the mean error (MEAN) of the tracking performance for subjects 1, 2, and 3 are 2.4°, 2.9°, and 3.4°; 2.2°, 2.8°, and 3.1°; and 2.1°, 2.6°, and 3.05°, respectively; the minimized mean square error (MSE) of the tracking performance for subject 1, 2, and 3 are 1.6°, 2.3°, and 2.6°; 1.3°, 1.7°, and 2.2°; and 1.4°, 1.9°, and 2.5°, respectively.

### 3.5. Case4: Assessment of Walk from up Slope to Level Ground

#### 3.5.1. Experiment

In this experiment, the subjects were required to walk from different slopes to level ground. The slope angles were varied from 7° to 5° and 3°, respectively. In the first 20 s, the subject walked on a slope at an angle of 7°. The slope angle gradually changed from 7° to 5° and then to 3°, each lasting for 20 s. The gait cycle was maintained at 2 s.

#### 3.5.2. Results

**Figure 7:**
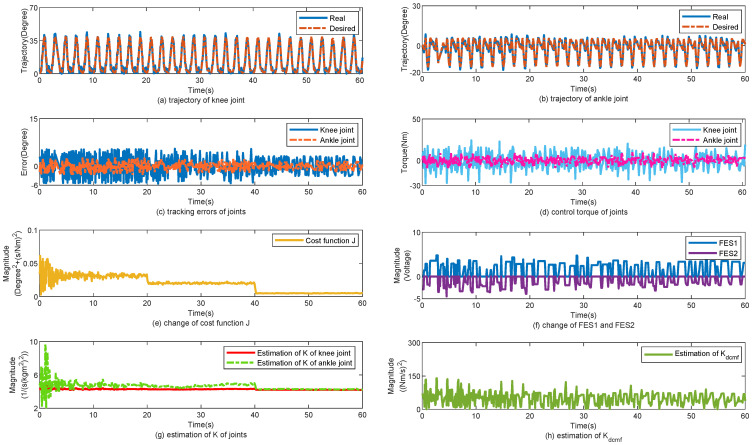
Walk from up slope to level ground. (**a**) Tracking performance on knee joint. (**b**) Tracking performance on ankle joint. (**c**) Tracking errors of joints. (**d**) Control torque of joints. (**e**) Change of cost function J. (**f**) Change of FES1 and FES2. (**g**) Estimation of K of joints. (**h**) Estimation of $K_{dcmf}$.

The control performance of the proposed method is illustrated in Figure 7. Figure 7a,b show the tracking trajectories of the knee and ankle joints, respectively. In the first 20 s, the slope angle was approximately 7°, and the gait cycle was approximately 2 s. The slope angle was then increased to 5° for 20 s. Finally, the slope angle was maintained at 3°. Figure 7c shows the tracking errors of the knee and ankle joints. With a decrease in the slope angle, the load on the joints was reduced, and the tracking errors of the joints were changed simultaneously. Figure 7d shows the change in the torques of the knee and ankle joint. Figure 7e shows the change in cost function *J*. According to (24), *J* is related to the tracking errors of joint *e*. When *e* is changed, *J* decreases simultaneously. Figure 7f shows the change in FES1 and FES2. Figure 7g,h show the changes in $\hat{K}$ and ${\hat{K}}_{dcmf}$, respectively. $\hat{K}$ decreased simultaneously with *J*. ${\hat{K}}_{dcmf}$ decreased with *J*. In Table 5, the tracking errors are statistically calculated for the three subjects. With the help of the proposed control method, the mean error (MEAN) of the tracking performance for subjects 1, 2, and 3 are 3.6°, 3.2°, and 2.5°; 3.5°, 3.0°, and 2.3°; and 3.2°, 3.4°, and 2.1°, respectively; the minimized mean square error (MSE) of the tracking performance for subjects 1, 2 and 3 are 3.1°, 2.8°, 2.1°, 2.6°, 2.7°, 1.4°, and 2.4°, 2.2°, 1.7°, respectively.

## 4. Discussion

Despite recent advances in the development of lower-limb prosthetics, there are advantages of restoring sensor feedback in controlling robotic leg prostheses for transtibial (below-knee) or transfemoral (above-knee) amputees. Functional Electrical Stimulation (FES) was widely used to reconstruct the sensations of prostheses. Most cases have established a sensory feedback loop by FES to feed back the movement of the leg to humans in real time. The restoration of sensory feedback could improve the walking performance of leg prostheses. However, the parameters of the FES were fixed and could not be adaptively regulated for walking terrain. Previous studies did not consider the cost function caused by contact force and noninvasive Functional Electrical Stimulation (nFES).

Hence, this study proposes a control strategy based on an extremum-seeking method to adjust the control parameters of the prosthetic leg to minimize the cost function. The extremum-seeking method was used to search for suitable control parameters as the cost function was changed. The proposed control method could be used to control different leg prostheses for both transtibial (below-knee) and transfemoral (above-knee) amputees. The main innovation lies in combining the extremum-seeking method with adaptive control to eliminate the need for manual tuning of control parameters. However, owing to the time required for optimization, when the walking terrain changes, the control method cannot immediately respond but still requires some time to change the control parameters.

## 5. Conclusions

The aim of this paper was to regulate the control parameters of a leg prosthesis based on changes in the tracking errors and stimulating current. The stimulating current was used to assist the subject in sensing changes in the contact force between the leg prosthesis and level ground. The cost function was designed by combining joint tracking errors and changes in the stimulating current. For different walking terrains, the extremum-seeking method (ESC) was used to search for suitable control parameters with a change in the cost function. The experimental results are discussed, and the effectiveness of the adaptive control framework is demonstrated. In Case 1, the mean error (MEAN) of the tracking performance is 3.6°, 3.3°, and 3.4°, respectively; the minimized mean square error (MSE) of the tracking performance is 2.3°, 2.8°, and 2.75°, respectively. In Case 2, the mean error (MEAN) of the tracking performance is 2.7°, 3.1°, and 3.2°, respectively; the minimized mean square error (MSE) of the tracking performance is 1.8°, 2.4°, and 2.3°, respectively. In Case 3, the mean error (MEAN) of the tracking performance for subjects 1, 2, and 3 is 2.4°, 2.9°, and 3.4°; 2.2°, 2.8°, and 3.1°; and 2.1°, 2.6°, and 3.05°, respectively; the minimized mean square error (MSE) of the tracking performance for subjects 1, 2, and 3 is 1.6°, 2.3°, and 2.6°; 1.3°, 1.7°, and 2.2°; and 1.4°, 1.9°, and 2.5°, respectively. In Case 4, the mean error (MEAN) of the tracking performance for subjects 1, 2, and 3 is 3.6°, 3.2°, and 2.5°; 3.5°, 3.0°, and 2.3°; and 3.2°, 3.4°, and 2.1°, respectively; the minimized mean square error (MSE) of the tracking performance for subjects 1, 2, and 3 is 3.1°, 2.8°, and 2.1°; 2.6°, 2.7°, and 1.4°; and 2.4°, 2.2°, and 1.7°, respectively. The experimental results demonstrated that the control parameters of the leg prosthesis could be regulated by changing the cost function in real time. The control scheme was mainly based on the extremum-seeking method to search for suitable control parameters for leg prostheses. Future research will focus on bipedal robotic leg prosthesis adaptive control and human-in-the-loop optimization.

## Figures and Tables

**Table 1:** Mass distribution of robotic prosthesis.

Part	Mass (kg)	Motion Range (°)
Body Structure	1.06	
Ankle Motor Unit	0.91	
Knee Motor Unit	1.01	
Electronics Assembly	0.38	
Motion Range of Knee		0° to 120°
Motion Range of Ankle		−45° to 45°
Total	4.8	

**Table 2:** Trajectory tracking performance in Case 1.

Subject	MEAN (Degree) with ECS	MSE (Degree) with ECS	MEAN (Degree) Without ECS	MSE (Degree) Without ECS
1	3.6	2.3	3.84	2.52
2	3.3	2.8	3.65	3.2
3	3.4	2.75	3.7	3.1

**Table 3:** Trajectory tracking performance in Case 2.

Subject	MEAN (Degree) with ECS	MSE (Degree) with ECS	MEAN (Degree) Without ECS	MSE (Degree) Without ECS
1	2.7	1.8	2.8	2.05
2	3.1	2.4	3.4	2.73
3	3.2	2.3	3.52	2.55

**Table 4:** Trajectory tracking performance in Case 3.

Subject	Up Slope Angle (Degree)	MEAN (Degree) with ECS	MSE (Degree) with ECS	MEAN (Degree) Without ECS	MSE (Degree) Without ECS
	3.0	2.4	1.6	2.8	1.9
1	5.0	2.9	2.3	3.3	2.5
	7.0	3.4	2.6	3.6	2.8
	3.0	2.2	1.3	2.5	1.7
2	5.0	2.8	1.7	3.2	2.1
	7.0	3.1	2.2	3.4	2.6
	3.0	2.1	1.4	2.7	1.8
3	5.0	2.6	1.9	3.1	2.2
	7.0	3.05	2.5	3.3	2.7

**Table 5:** Trajectory tracking performance in Case 4.

Subject	Up Slope Angle (Degree)	MEAN (Degree) with ECS	MSE (Degree) with ECS	MEAN (Degree) Without ECS	MSE (Degree) Without ECS
	7.0	3.6	3.1	3.8	3.5
1	5.0	3.2	2.8	3.6	3.3
	3.0	2.5	2.1	2.7	2.35
	7.0	3.5	2.6	3.7	2.9
2	5.0	3.0	2.7	3.3	3.05
	3.0	2.3	1.4	2.55	1.65
	7.0	3.2	2.4	3.5	2.6
3	5.0	3.4	2.2	3.65	2.35
	3.0	2.1	1.7	2.35	1.9

## Data Availability

Data are contained within the article.
